# The immunological landscape in necrotising enterocolitis

**DOI:** 10.1017/erm.2016.13

**Published:** 2016-06-24

**Authors:** Steven X. Cho, Philip J. Berger, Claudia A. Nold-Petry, Marcel F. Nold

**Affiliations:** 1 Ritchie Centre, Hudson Institute of Medical Research, Melbourne, Australia; 2 Department of Paediatrics, Monash University, Melbourne, Australia

## Abstract

Necrotising enterocolitis (NEC) is an uncommon, but devastating intestinal inflammatory disease that predominantly affects preterm infants. NEC is sometimes dubbed the spectre of neonatal intensive care units, as its onset is insidiously non-specific, and once the disease manifests, the damage inflicted on the baby's intestine is already disastrous. Subsequent sepsis and multi-organ failure entail a mortality of up to 65%. Development of effective treatments for NEC has stagnated, largely because of our lack of understanding of NEC pathogenesis. It is clear, however, that NEC is driven by a profoundly dysregulated immune system. NEC is associated with local increases in pro-inflammatory mediators, e.g. Toll-like receptor (TLR) 4, nuclear factor-κB, tumour necrosis factor, platelet-activating factor (PAF), interleukin (IL)-18, interferon-gamma, IL-6, IL-8 and IL-1β. Deficiencies in counter-regulatory mechanisms, including IL-1 receptor antagonist (IL-1Ra), TLR9, PAF-acetylhydrolase, transforming growth factor beta (TGF-β)_1&2_, IL-10 and regulatory T cells likely facilitate a pro-inflammatory milieu in the NEC-afflicted intestine. There is insufficient evidence to conclude a predominance of an adaptive Th1-, Th2- or Th17-response in the disease. Our understanding of the accompanying regulation of systemic immunity remains poor; however, IL-1Ra, IL-6, IL-8 and TGF-β_1_ show promise as biomarkers. Here, we chart the emerging immunological landscape that underpins NEC by reviewing the involvement and potential clinical implications of innate and adaptive immune mediators and their regulation in NEC.

## Introduction

Necrotising enterocolitis (NEC) is a serious gastrointestinal disease that most commonly afflicts infants born prematurely. Although infrequent, NEC is a major cause of morbidity and mortality in neonatal intensive care units (NICUs). In older children, NEC occurs most commonly in association with cyanotic heart disease or major cardiac surgery (Ref. 1). NEC is a multifactorial disease whose pathogenesis remains poorly understood despite decades of research. However, risk factors for NEC have been identified, namely prematurity, formula feeding, hypoxic–ischaemic injury and abnormal bacterial colonisation. Yet, no single risk factor is essential, and the mechanisms by which each precipitates NEC are largely unknown. Nonetheless, evidence is mounting that formula feeding, hypoxia–ischemia, and dysbiosis lead to inflammation, and that immaturity of the immune system in preterm babies – although itself poorly characterised – is one of the pivotal pathogenic factors in NEC. Here, we review current knowledge on inflammation and immunity in NEC and highlight frontiers emerging in this field.

## Epidemiology, staging criteria and disease outcomes

Death of extremely premature infants from most causes has decreased across the period from 2000 to 2011, whereas the incidence of death from NEC has increased (Ref. 2). Thus, NEC is now the most common cause of death between days 15 and 60 (Ref. 2). The overall incidence of NEC is 1–3 per 1000 live births (Ref. 3), but reaches 11% in very low birth weight infants (VLBW, <1500 g) (Ref. 4). NEC-associated mortality has changed little over the past 50 years, ranging from 20 to 30% in confirmed cases (Ref. 5). Approximately 20–50% of NEC infants require surgery; mortality then rises to about 65% (Refs 4, 6, 7).

**Figure 1.**
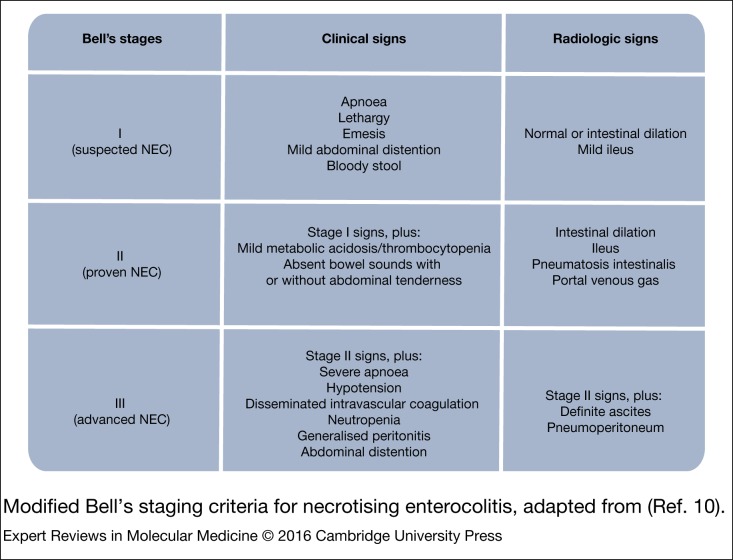
Modified Bell's staging criteria for necrotising enterocolitis, adapted from (Ref. 10).

Treatment options for NEC infants are limited to bowel rest, antibiotics and supportive therapy, e.g. blood pressure management (Ref. 8). Decisions on such treatment or escalation to surgery are aided by Bell's staging criteria (Refs 9, 10) (Fig. 1). The clinical presentation of stage I NEC is largely non-specific, which explains why diagnosing NEC early is difficult. It is for this reason, and because NEC often manifests rapidly and quickly wreaks intestinal and systemic havoc that many neonatologists perceive NEC as an ever-looming spectre in NICUs.

Short-term consequences of NEC include severe multisystem morbidity, leading to extended hospitalisation with all its financial and social burdens (Ref. 11). The cost of surgically managed NEC is enormous at approximately US$200,000 per survivor *in excess* of the per-baby cost of routine neonatal intensive care (Refs 11, 12).

In childhood, prior history of NEC is an independent risk factor for bowel-related chronic conditions such as diarrhoea and constipation (Ref. 13). Similarly, neurodevelopmental issues often persist into later life and may include epilepsy, attention deficit hyperactivity disorder, cerebral palsy, deafness, blindness and compromised mental and psychomotor functions (Refs 13, 14, 15). Half of all surgically managed NEC infants develop some degree of short-bowel syndrome/intestinal failure (Ref. 16), and poor growth is common, particularly in extremely low birth weight (ELBW, <1000 g) NEC infants (Ref. 15).

## NEC pathogenesis and risk factors

### Prematurity

**Figure 2.**
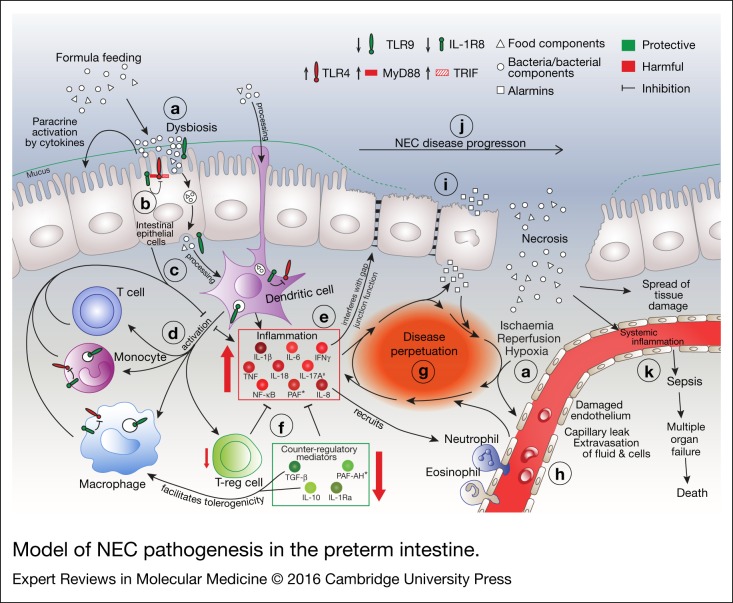
Model of NEC pathogenesis in the preterm intestine. (a) Multiple factors are involved in the precipitation of NEC, including dysbiosis, formula feeding, and ischaemic/hypoxic assaults. (b) Inappropriate increases in abundance of, and signalling by, pro-inflammatory pattern recognition receptors (PRRs) such as TLR4 contribute to the initiation of a cascade that involves (c) antigen processing by antigen-presenting cells such as dendritic cells (DCs) and (d) activation of other immune cells such as T cells, monocytes, macrophages and regulatory T cells (Tregs), leading to (e) an inappropriate and excessive increase of pro-inflammatory cytokines, chemokines and transcription factors. (f) A deficiency in counter-regulatory mediators contributes to this pro-inflammatory milieu to self-perpetuate and spiral out of control – (g) a vicious cycle is formed. (h) Inflammation-, ischaemia/reperfusion- and hypoxia-associated injury compromises the endothelial integrity of the local blood vessels, which also feeds the vicious cycle. (i) Necrotic cell death of the intestinal epithelium ensues, further exacerbating tissue injury and inflammation. (j) In line with the clinical stages (see Fig. 1), NEC severity can range from mild intestinal injury to segmental or even complete destruction of the intestinal epithelium. (k) Disintegration of the intestinal epithelium compromises its barrier functions, ultimately leading to rampant bacterial translocation into the lamina propria and the systemic circulation. Sepsis, multi-organ failure and death ensue. *, systemic data. #, strong evidence to be harmful only from one paper.

NEC incidence and severity are most strongly associated with prematurity, quantified either as low gestational age (GA) or low weight at birth (Refs 17, 18, 19). Briefly, NEC may arise on the basis of the interactions between two poorly developed systems, namely the intestine and the immune system (Refs 20, 21, 22) (Fig. 2). Immaturity of intestinal motility and mucosal/barrier functions facilitates a potentially harmful composition of the microbiome and bacterial translocation (Fig. 2a). Thus confronted with bacteria, the premature immune system responds by unleashing a violent inflammatory storm (Fig. 2e) that overwhelms the extant endogenous counter-regulatory mechanisms (Fig. 2f), leading to cell death and subsequent release of intracellular components such as stored cytokines termed alarmins (Fig. 2i) (Ref. 23), thus perpetuating the inflammatory storm (Fig. 2g). As described below in detail, a poorly controlled, excessive inflammatory response is one of the major factors that not only triggers the cascade that ultimately leads to NEC, but also maintains disease activity as part of a vicious cycle (Fig. 2g).

### Formula feeding

Formula feeding is a well-established risk factor for NEC (Fig. 2a), and the incidence of NEC in infants fed their own mother's milk is reduced compared with formula-fed infants (Ref. 24). Exclusive feeding with their own mother's milk was also associated with fewer episodes of late-onset sepsis and/or NEC (OR 0.18; 95% CI 0.04–0.79, *P* = 0.02) and shorter duration of hospital stay compared with formula- or donor breast milk-fed infants (Ref. 25). A meta-analysis of studies comparing formula with donor breast milk in preterm or LBW infants revealed that formula triples the risk of NEC (Ref. 26). Infant formula contains components such as unbound free fatty acids (Ref. 27) that may facilitate NEC, and is deficient in potentially protective factors such as anti-inflammatory cytokines, immunoglobulins, growth factors, and microbiota, which are present in breast milk (Refs 28, 29). Further details are discussed in the relevant sections below.

### Hypoxia–ischaemia

Historically, intestinal hypoxic–ischaemic injury was considered the single most important factor initiating and perpetuating NEC, a view consistent with the predominant pathologic finding being coagulative necrosis, a common sequela of prior ischaemia (Ref. 30). In addition, term neonates with NEC often have conditions such as chronic heart disease that favour hypoxic or ischaemic states (Fig. 2a) (Refs 31, 32). However, no primary hypoxic–ischaemic event can be identified in most preterm infants presenting with NEC. The appearance of NEC at 2–3 weeks of age (Ref. 33) (when pronounced or prolonged hypoxia/ischaemia is uncommon) rather points to a role of intestinal bacterial colonisation, which is usually nearly complete by this time.

### Microbial colonisation

The gut microflora plays an important role in regulating gut immune homeostasis, e.g. by dampening excessive inflammatory responses and establishing an environment ‘tolerogenic’ for commensal bacteria (Ref. 34). This dampening process may be disrupted in NEC because of lower microflora diversity compared with preterm controls (Ref. 35). It is currently unclear whether the dysbiosis (Fig. 2a) that often accompanies NEC is a consequence or one of the causes of abnormal immune interactions between gut bacteria and the preterm intestine. Nevertheless, the role of the initial microbial colonisation in NEC is probably important as experimental NEC does not develop in the absence of bacteria, i.e. in germ-free piglets (Ref. 36) or mice treated with antibiotics (Ref. 37).

Of note, animal studies have implicated *Clostridium butyricum* in NEC (Refs 38, 39, 40), and a recent study in human infants found this bacterium in the stool of 80% of NEC infants compared with 12% of controls (Ref. 41). Although these findings are promising, it is too early to conclude that *C. butyricum* is a bacterial cause of NEC.

## Animal models of NEC

Much of our understanding of NEC pathogenesis stems from animal models of the disease, with the majority using rats, mice or piglets [reviewed in (Ref. 42)]. Most published models employ one or several of the known risk factors that induce NEC-like intestinal injury. The earliest NEC model, dating to 1974, subjected newborn rats to formula feeding and hypoxic stress (Ref. 43). This model is still used today, the most common variant being to subject caesarean-born preterm rats to formula feeding, hypoxia and hypothermia. Other variants of the hypoxia–hypothermia model include using caesarean-born E18.5 mice (Ref. 44), naturally delivered newborn mice (Ref. 45) and 7–10-day-old mice (Ref. 46). As newborn mice are more difficult to feed and handle than rats, the variant using 10-day-old mice is widely used today. Less commonly, murine NEC is induced using 2,4,6-trinitrobenzene sulphonic acid by gavage or enema in 10-day-old mice (Ref. 47), ablation of Paneth cells in combination with gavage feeding of *Klebsiella* in 14–16-day-old mice (Ref. 48), and by oral administration of *Cronobacter sakazakii* in 3-day-old mice (Ref. 49). Rabbit and hamster NEC models are occasionally employed, but most large animal-work on NEC is conducted in piglets. The gastrointestinal tract of newborn piglets closely resembles that of human babies in terms of anatomy, physiology, development and function. Piglet NEC models commonly comprise preterm birth, parenteral nutrition and formula feeding, but no exposure to hypoxia or hypothermia. NEC can also be modelled in primates, but such research is rarely undertaken as it requires preterm delivery and care for weeks in a NICU-like setting (Ref. 50). Another rare model is the gnotobiotic quail, primarily used for investigation of the role of clostridia in NEC (Refs 39, 51).

## NEC and Immunity

**Figure 3.**
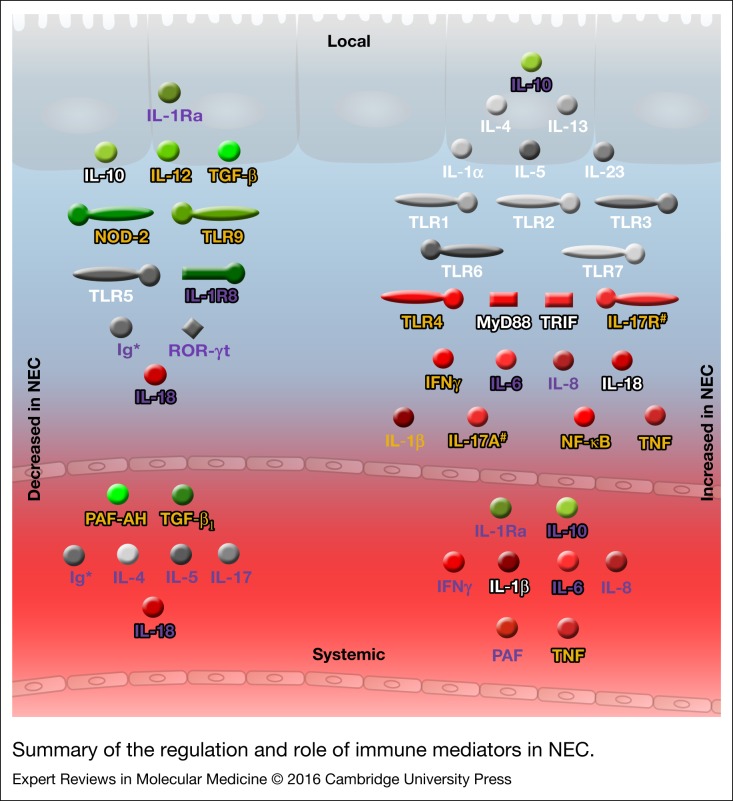
Summary of the regulation and role of immune mediators in NEC. Green, protective; Grey, inconclusive; and Red, harmful. White text, animal data; purple text, human data; yellow text, animal and human data; black text outline, functional and/or genetic data. *Ig*, immunoglobulin; *IFNγ*, interferon gamma; *IL*, interleukin; *IL-1Ra*, interleukin-1 receptor antagonist; *IL-1R8*, IL-1 receptor 8; *IL-17R*, IL-17 receptor; *MyD88*, myeloid differentiation factor 88; *NF-κB*, nuclear factor-κB; *NOD-2,* nucleotide-binding oligomerisation domain-containing protein 2; *PAF,* platelet-activating factor; *PAF-AH*, PAF-acetylhydrolase; *RORC*, RAR-related orphan receptor C; *TNF*, tumour necrosis factor; *TRIF*, toll/IL-1R domain containing adaptor inducing IFNβ; *TLR*, toll-like receptor; *TGF-β*, transforming growth factor beta; *, may be protective in NEC, but Ig supplementation has not proven effective; #, strong evidence to be harmful only from one paper.

The relationship between the immature preterm immune system and NEC is complex. A number of innate and adaptive immune mediators have been implicated in NEC, as summarised in Figure 3; note the distinction between local and systemic events. It is also important to keep in mind that evidence from human resection specimens is virtually always obtained from advanced NEC stages; therefore, knowledge on the intestinal events occurring in early human NEC is all but non-existent.

## The immune system in preterm neonates

Detailed discussion of this topic is beyond the scope of this review, but briefly: The immune system is divided into two arms, innate and adaptive immunity. The newborn relies predominantly on innate immunity during early life as maturation of adaptive immunity lags behind that of innate immunity (Ref. 52). Within the adaptive arm, type 2 T-cell polarisation predominates in mother and foetus, thus protecting both from graft-versus-host-type rejections, which are mediated by type 1-polarised responses (Ref. 53). Compared with term infants, other differences include lower immune cell counts (Ref. 54), lower expression of major histocompatibility class II molecules (Ref. 55), and reduced phagocytic ability of monocytes and neutrophils (Ref. 56).

## Innate immunity

The innate arm of immunity is phylogenetically older than adaptive immunity and functions as the first line of defence against potential pathogens. Innate immunity has two key components; a static component that consists of epithelial surfaces such as the skin and the gastrointestinal epithelium, which serve as physical barriers against microbial entry, and a reactive component, which involves tissue-resident and patrolling immune cells that are poised to respond rapidly to potential threats.

## Pattern recognition receptors (PRRs)

PRRs play a central role in innate immunity, as they recognise pathogen-associated molecular patterns of invading pathogens and initiate signalling cascades that lead to target-independent inflammatory responses. As they are expressed by most cell types, PRRs perform a key function in frontline surveillance (Ref. 57). Two families of PRRs, Toll-like receptors (TLRs) and Nod-like receptors (NLRs), have been implicated in NEC.

## Toll-like receptors

In the intestine, TLRs are expressed by immune cells and intestinal epithelial cells (IECs) (Ref. 58). A fine balance is required between preventing tissue invasion by gut bacteria on the one hand and establishing tolerance of a luminal commensal, symbiotic gut flora on the other. Therefore, the function of TLRs must be tightly controlled, particularly during the transition of the newborn gut from a germ-free intrauterine environment to postnatal exposure to colonising bacteria. Of note, much of our knowledge on TLRs in NEC stems from animal experiments, and it should be kept in mind that animal and human data are not always congruent.

### TLR4

Among the TLRs, TLR4 has received by far the most attention in the context of NEC. TLR4 is activated by the Gram-negative bacterial cell wall component lipopolysaccharide (LPS), a prototypical trigger of inflammation. Abundance and function of TLR4 is tightly regulated: Late in murine pregnancy (up to day 18; normal duration 21 days), *Tlr4* mRNA expression increases, but rapidly decreases immediately following birth, thus adapting innate responses to the new environment (Ref. 59). Functionally, murine foetal IECs are significantly more responsive to LPS than IECs isolated on postnatal days 1 and 6 (Ref. 60). Xenografts from more immature human foetal ileum also express 3-fold more *TLR4* than more mature grafts when transplanted into SCID (severe combined immunodeficiency) mice (Ref. 61).

TLR4 gene and protein expression are elevated in the small intestinal mucosa of both human and mouse NEC compared with healthy controls (Fig. 2b) (Refs 59, 62, 63). This important signalling node is also target of mediators in breast milk such as soluble CD14, lactadherin, lactoferrin and 2′-fucosyllactose (Ref. 64). In a study in which lactating mice were milked under anaesthesia, mouse breast milk attenuated murine NEC by reducing TLR4 signalling, and overexpression of TLR4 in the intestinal epithelium reverses these protective effects (Ref. 65). In mice, excessive TLR4 expression was moreover linked to inhibition of intestinal repair, via activation of the p53-up-regulated modulator of apoptosis (Ref. 66) as well as induction of endoplasmic reticulum (ER) stress in intestinal stem cells (Ref. 67). Increased ER stress and apoptosis have been observed in the intestinal crypts of human NEC patients (Ref. 67).

A pathogenic role of TLR4 in NEC appears likely, as TLR4-deficient mice (Ref. 37) and mice with non-functional TLR4 (Ref. 63) were protected against NEC-associated tissue damage, and a small molecule TLR4 inhibitor (C34) administered by oral gavage reduced ileal NEC injury (Ref. 68). Interestingly, enterocyte-specific deletion of TLR4 also efficiently protected from NEC, suggesting that the epithelium participates in this aspect of the disease (Ref. 37). Indeed, there is evidence that TLR4 expression in the intestinal epithelium may influence the recruitment and polarisation of T cells in the intestinal mucosa (Ref. 69).

### TLR9

Interestingly, TLR9, which recognises the characteristically CpG-rich bacterial DNA, acts as a counter-regulator of the disease-promoting effects of TLR4 in NEC (Ref. 59). Regulation of *Tlr9* gene expression in the murine ileum is opposite to that of TLR4, so that *Tlr9* decreases during late pregnancy, but increases at birth (Ref. 59). Mouse pups receiving two injections of 1 mg/kg CpG-DNA per day (Ref. 59) or once-daily oral CpG-DNA (Ref. 46) exhibited reduced NEC severity compared to vehicle-treated pups, demonstrating a functional relevance for TLR9 in NEC. Conversely, a mutation rendering TLR9 unresponsive to CpG-DNA causes increased NEC severity in mice (Ref. 59). Similarly, *Lactobacillus rhamnosus*-mediated protection in murine NEC is also dependent on TLR9 activation, as protection was abolished upon selective lentiviral knockdown of intestinal epithelial TLR9 (Ref. 46). A small human study showed that TLR9 protein abundance was reduced in NEC patients compared with controls (Fig. 2b) (Ref. 59); however, a protective function of TLR9 has not been confirmed in humans.

### TLR5 and other TLRs

Gene expression of *Tlr1, -2, -3, -6* and -*7* was increased in ileal tissue of NEC rats compared with dam-fed controls, with only *Tlr5* decreased (Refs 70, 71). A NEC-associated decrease in *Tlr5* is consistent with TLR5 knockout mice developing spontaneous colitis (Refs 72, 73). The underlying mechanism between decreased TLR5 and chronic intestinal inflammation remains unknown, but it was speculated that absence of epithelial TLR5 may reduce epithelial barrier functions and thus increase bacterial translocation (Ref. 72). Alternatively, the decrease in *Tlr5* mRNA may be secondary to increased TLR2 and -4 activation (Ref. 74); notably, *Tlr2* and *-4* are elevated in resected intestinal tissue from infants with stage III NEC (Ref. 62).

In summary, aberrantly elevated TLR4 signalling has a pathogenic role in NEC, whereas TLR9 and possibly TLR5 act as counter-regulators of TLR4. The functional relevance of other TLRs in the disease remains poorly defined.

## Nucleotide-binding oligomerisation domain (NOD)-like receptors

NLRs are intracellular PRRs and are critical mediators of the assembly of the inflammasome, which converts the pro-forms of the pro-inflammatory cytokines interleukin (IL)-1β and IL-18 into their mature, active forms. Data on NLRs in NEC are scant.

### NOD-2

NOD-2 is a sensor of bacterial cell-wall fragments, specifically muramyl dipeptide (MDP). NOD-2 mediates production of anti-bacterial defensins in epithelial Paneth cells (Ref. 75) and elicits immune responses through the nuclear factor (NF)-κB pathway (Ref. 76). NOD-2 activity may exert protective effects in NEC as daily injections of MDP almost completely abolished NEC-associated intestinal tissue damage in mice (Ref. 77). Similarly, in humans, NOD-2 loss-of-function mutations has been associated with Crohn's disease (CD) (Refs 78, 79) and VLBW infant carriers of two or more NOD-2 loss-of-function alleles had an increased risk for NEC requiring surgery (OR 3.57; 95% CI 1.3–10.0, *P* = 0.03) (Ref. 80).

## Mediators of innate immunity

### IL-1

IL-1 is the prototypical pro-inflammatory cytokine, and is induced in numerous cell types by a wide variety of triggers. Active at picogram concentrations, IL-1 induces a plethora of inflammatory effects, including the production of other pro-inflammatory mediators, tissue damage and fever (Ref. 81). The two isoforms, IL-1α and IL-1β, bind to the same heterodimeric cell surface receptor (Ref. 81). Activation and release of IL-1β are tightly controlled by post-translational mechanisms such as processing by caspase-1, which in turn is regulated by the inflammasome. Therefore, data on *IL1B* mRNA not accompanied by protein measurements may not be indicative of biological activity and should be interpreted with great caution. IL-1 binding to its receptor triggers a signalling cascade that results in activation of pro-inflammatory transcription factors such as NF-κB and AP-1, which in turn induce pro-inflammatory cytokines such as IL-6, tumour necrosis factor (TNF) and IL-1 itself (Ref. 81).

Studies on IL-1α in NEC are rare. In caesarean-delivered preterm piglets with NEC, lysates of the small intestine exhibited increased *IL1A* mRNA abundance compared to colostrum-fed controls (Refs 82, 83). This increase in *IL1A* expression was rapid, occurring at 8 h and persisting for up to 34 h post-NEC induction (Ref. 82).

IL-1β protein was elevated systemically (Ref. 84) and in intestinal tissue in animal models of NEC (Fig. 2e) (Refs 70, 85). In newborn rats, 48 h of formula feeding alone increased IL-1β protein in the terminal ileum 3-fold compared with dam-fed controls (Ref. 70). Induction of NEC increased IL-1β up to 6-fold compared with dam-fed controls (Refs 70, 85). Importantly, the authors highlighted that increases in IL-1β preceded tissue injury, which did not occur before 72 h (Ref. 70).

In one of the few human studies on IL-1β in NEC, ileal *IL1B* mRNA in surgical NEC infants was more than 10-fold higher compared with GA-matched non-NEC controls (Ref. 86). Similarly, *in situ* hybridisation experiments showed a more than 2-fold increase in *IL1B* mRNA in full-thickness sections of stage III NEC infants compared with surgical controls (Ref. 87). Systemically, there was no difference between the pre-operative serum IL-1β abundance in NEC babies and non-NEC controls (Ref. 88). Similarly, limited time course experiments in human NEC infants beginning at NEC onset (defined by a combination of clinical and laboratory findings) and covering 8, 24, 48 and 72 h showed no significant change in serum IL-1β (Ref. 89). However, there was a trend towards higher IL-1β abundance in stage III infants compared with stage I and II infants (Ref. 89).

Overall, the available data indicate that increased IL-1 precedes NEC injury, suggesting that IL-1 aggravates tissue damage and contributes to NEC initiation and perpetuation of the vicious cycle (Fig. 2g).

### IL-1 receptor antagonist (IL-1Ra)

IL-1Ra is an anti-inflammatory cytokine that functions by competitively inhibiting the binding of the two pro-inflammatory ligands IL-1α and IL-1β to their receptor. IL-1Ra is in clinical use as reviewed in (Ref. 81), though at present not in NEC.

As IL-1Ra is one of the endogenous counter-regulatory mechanisms induced by inflammation, its abundance is often associated with disease severity in inflammatory diseases. However, the considerable increases in IL-1Ra observed in NEC (Ref. 89) clearly do not curtail the overwhelming inflammation that underpins NEC; perhaps IL-1Ra concentrations are insufficiently elevated in the gut where the inflammatory damage is occurring. Interestingly, IL-1Ra was decreased 2–3 weeks prior to NEC onset in buccal swabs from at-risk infants (Ref. 90), suggesting a causative connection between NEC and IL-1Ra deficiency (Fig. 2f). Indeed, IL-1Ra shows promise as a NEC biomarker as described below.

### Tumour necrosis factor

TNF, like IL-1, is a key pro-inflammatory cytokine that activates inflammatory mediators such as NF-κB in virtually any cell type.

TNF was increased systemically (Ref. 91) and in intestinal tissue (Ref. 92) of NEC patients compared with non-NEC controls (Fig. 2e), but was not indicative of disease severity (Refs 88, 93, 94, 95). Ileal and systemic TNF were also increased in rat models of NEC (Refs 96, 97, 98), with the mRNA rising as early as 1.5 h after the first feed (Ref. 99). Although others did not observe such increases in TNF (Ref. 100), functional data indicate a disease-promoting role for TNF. Inhibition of TNF via administration of a monoclonal anti-TNF antibody (Refs 98, 101), pentoxiphylline (Ref. 102), etanercept (Ref. 103) or infliximab (Ref. 104) significantly reduced intestinal inflammation and tissue injury in neonatal NEC rats. However, others have reported no significant improvement with pentoxiphylline in hypoxia/reperfusion-induced rabbit NEC (Ref. 105).

These observations suggest that TNF contributes to NEC progression, likely with a major role in the early stages of the disease. The usefulness of TNF as a biomarker in NEC appears limited.

### IL-6

IL-6 is an important acute phase immune mediator; for example, it stimulates hepatocytes to produce acute-phase proteins such as C-reactive protein (CRP). In fact, both CRP and IL-6 are in clinical use as biomarkers of acute inflammation (Ref. 106).

It is likely that excessive IL-6 plays a pathogenic role in NEC. Genetic analysis of IL-6 single nucleotide polymorphisms (SNPs) in neonates of 32 weeks gestation or less revealed that Caucasians with IL-6 rs1800795, an SNP that is associated with increased plasma IL-6 in neonates (Ref. 107), were six times more likely to develop NEC and seven times more likely to progress to stage III disease (Ref. 108). These observations agree with studies that demonstrated elevated IL-6 protein (Ref. 109) and mRNA expression (Refs 62, 110) in resected intestinal tissue of stage III NEC patients compared with controls (Fig. 2e). IL-6 may thus be useful as a biomarker in NEC; see the Biomarkers section.

### IL-10

IL-10 is an important dampener of immune responses in the intestine, and loss of IL-10 or its receptor (IL-10R) results in early-onset inflammatory bowel disease in humans (Ref. 111) and mice (Ref. 112). Although the interaction between the intestinal microbiome and immunity is not part of this review, it is interesting to note that the intestinal inflammation of IL-10-deficient mice does not develop in a pathogen-free environment (Ref. 112).

IL-10 functionality in macrophages curtails intestinal inflammation, as specific knockout of IL-10R signalling in intestinal lamina propria-resident macrophages results in severe spontaneous colitis in mice (Ref. 113). The number of regulatory T cells (Treg), an important source of intestinal IL-10 (Ref. 114), was reduced in the ileum of NEC rats compared to dam-fed controls (Fig. 2f) (Ref. 115). Similarly, in humans, the total number of CD4^+^Foxp3^+^ Treg and the Treg/T effector ratio was reduced in the lamina propria of surgical NEC infants compared to surgical controls (Ref. 86). Mice deficient in IL-10 exhibited more severe epithelial damage and overall NEC injury than wild-type controls (Fig. 2f) (Ref. 116). Moreover, administration of exogenous IL-10 to IL-10-deficient mice prior to NEC induction prevented mucosal injury (Ref. 116). IL-10 as a protective factor in NEC is supported by the observation that human breast milk contains high concentrations of bioactive IL-10 (Ref. 117) and lower IL-10 abundance in breast milk correlates with increased human NEC incidence (Ref. 118).

However, a deficiency in IL-10 is not observed in human NEC; indeed, both serum and ileal IL-10 were markedly increased in infants diagnosed with NEC, particularly in those with advanced NEC (Refs 86, 88, 89), which, as with IL-1Ra, is likely part of the immune system's inadequate attempt at countering the excessive inflammation. As NEC predominantly affects preterm infants, it should also be noted that prematurity does not predispose to IL-10 deficiency (Refs 119, 120) or inducibility by TLR agonists (Refs 119, 121, 122).

It thus appears likely that IL-10 contributes to dampening inflammation in NEC, but its precise role in NEC pathogenesis remains unclear.

## Mediators of innate immune signalling

### Nuclear factor-κB

NF-κB is the prototypical pro-inflammatory transcription factor, with many pathways converging at this central node of inflammatory signalling. TLR-, IL-1 receptor (IL-1R)-, and TNFR-activation trigger a cascade that leads to release of cytoplasmic NF-κB from its inhibitory protein, the inhibitor of κB (IκB), allowing NF-κB to translocate to the nucleus and to actuate the transcription of pro-inflammatory mediators, including cytokines, chemokines and leukocyte adhesion molecules (Ref. 123). Developmental regulation of NF-κB pathway components may favour NEC, e.g. a reduced abundance of IκB in foetal primary IEC compared with mature adult enterocytes (called T84 cells) (Ref. 124).

In animals, vaginal birth may trigger a transient, low-grade increase in NF-κB activation in the small intestine, possibly allowing a tolerogenic immune surveillance of the early stages of bacterial colonisation (Ref. 60): NF-κB was activated in murine IECs as early as 60 min after natural birth in the absence of inflammatory stimuli (Ref. 60) before its activation returned to baseline by 24 h (Ref. 99). Conversely, NF-κB activity was nearly undetectable in the small intestine of newborn rats delivered by caesarean section (Ref. 125). These findings may contribute to the unexpected observation that vaginal birth is a risk factor for early onset NEC (defined as <14 days, stage II or higher) in human preterm infants of <33 weeks GA (Ref. 126). However, the association between vaginal birth and intestinal NF-κB activation has not been demonstrated in human infants.

On the other hand, there is clear evidence for an involvement of NF-κB in NEC. First, NEC severity was correlated with increased NF-κB activity in the epithelial cells of caesarean-born pups (Fig. 2e) (Refs 71, 99, 125), and second, specific inhibition of NF-κB (using a NEMO-binding domain peptide) in NEC rats markedly reduces disease incidence and severity (Ref. 125). Furthermore, in a human study, 100% of NEC infants were carriers of the *NFKB1* variant –94delATTG, which leads to more pronounced inflammatory responses to LPS (Ref. 127), compared to 65% of the non-NEC infants (Ref. 128).

### MyD88 (myeloid differentiation factor 88), TRIF [Toll/IL-1R domain containing adaptor inducing interferon (IFN)β] and IL-1R8 (IL-1 receptor 8, previously called SIGIRR)

The first step in the TLR- and IL-1R signalling cascades is recruitment of adapter molecules to the intracellular domains of the receptors. For example, TLR4 activates two signalling pathways, one via the adapter MyD88 and one via TRIF (Ref. 129).

In concordance with the finding that TLR4-deficient mice were protected from NEC injury (Ref. 37), deficiency in MyD88 (Ref. 130) and TRIF (Ref. 37) also attenuated the disease (Fig. 2b). Unexpectedly, the protection conferred by the absence of MyD88 was not as complete as that observed in mice deficient in TLR4 and TRIF, indicating an important role for TRIF-dependent signalling in NEC (Ref. 37). Similarly, a deficiency in IL-1R8, which is a negative regulator of TLR- and IL-1R signalling (Refs 131, 132), may also be important as a small study associated NEC infants with stop-, missense- or splice region-IL-1R8 variants (Fig. 2b) (Ref. 133).

## Adaptive immunity

The immune system's adaptive arm responds to highly specific antigens, which must be processed and presented, again in a highly specific fashion, by antigen-presenting cells (APC). The prototypic APC are dendritic cells (DC), which present antigens to T and B cells, the major effector cells of adaptive immunity. Such presentation results in the polarisation of naïve CD4^+^ T helper (Th) cells into different subsets, including Th1, Th2, Th17 and Treg, with the subset determination depending on the state of the APC, the antigen, its presentation, and the local cytokine milieu. Each subset is characterised by predominance of a transcription factor (T-bet, GATA-3, Ror-γt and Foxp3, respectively) and signature cytokines (IFNγ, IL-4, IL-17A and IL-10, respectively). Generally, the subsets antagonise each other, e.g. Th1 cytokines inhibit Th2 polarisation.

There are conflicting data on the lymphocyte fraction of the inflammatory tissue infiltrate in NEC: Whereas a lamina propria CD4^+^ T cell component of 30–40% in NEC mouse pups and human infants was reported (Ref. 69), others observed a paucity of lymphocytes in the inflammatory infiltrate in human NEC infants (Refs 47, 134). Thus, the data discussed below need to be interpreted with caution. Nevertheless, some animal studies provide evidence to support a role for CD4^+^ T cell influx as an important pathogenic event in NEC. For example, recombination activating gene-deficient (Rag1^−/−^) mice, which are deficient in functional T and B cells, exhibit significantly reduced NEC-associated intestinal injury and *Il1b* expression compared with wild-type controls (Ref. 69). In addition, adoptive transfer of naïve CD4^+^ T cells to Rag1^−/−^ mice prior to NEC induction restored susceptibility to severe NEC (Ref. 69). Furthermore, transfer and repopulation of Rag1^−/−^ mice with CD4^+^ T cells from wild-type mice with NEC led to intestinal damage and increased *Il1b* expression after 48 h (Ref. 69). RNA sequencing of ileal samples from surgical NEC infants also revealed strongly altered T and B cell signalling in NEC compared with non-NEC preterm controls (Ref. 135). Although surprisingly little information is available on the role of Th subsets in initiation and/or perpetuation of NEC, some of the signature cytokines have been investigated.

## Th1 Cytokines

### IFNγ

IFNγ is the signature cytokine of Th1 immune responses. It contributes to the differentiation of Th1 cells and exerts pro-inflammatory actions by inducing Th1 chemokines, activating macrophages and facilitating phagocytosis (Ref. 136). The combined effects of IFNγ are critical to clearance of intracellular pathogens. Of note, prematurity is associated with a reduced capacity to mount Th1 responses and produce IFNγ (Ref. 137).

Whereas one human study reported no difference between peri-operative serum IFNγ in NEC infants and non-NEC controls (Ref. 88), others found a 4-fold higher frequency of cells spontaneously secreting IFNγ in peripheral blood mononuclear cells (PBMCs) isolated from stage II and III NEC infants at diagnosis compared with age-matched healthy controls (Ref. 138). Similarly, contradictory observations were made on *IFNG* mRNA in intestinal resection specimens (Refs 139, 140).

In rats and mice, the data more clearly point to a disease-promoting role for IFNγ, as ileal IFNγ protein abundance dramatically increased after induction of experimental NEC compared with dam-fed controls (Fig. 2e) (Refs 70, 141). Mechanistically, excessive IFNγ interferes with epithelial barrier integrity and regeneration, including function of intercellular gap junctions and IEC migration (two processes impaired in wild-type NEC mice but unaffected in IFNγ-deficient NEC mice) (Ref. 141). Abrogation of these detrimental effects of IFNγ is likely to contribute to the observation that 10-day-old IFNγ-deficient mice are completely protected from NEC-associated ileal tissue damage (Ref. 141).

### IL-12

The principal function of IL-12 is to promote and maintain Th1 polarisation, for example by induction of IFNγ. Animal studies of NEC are inconclusive about IL-12, one reporting lower (Ref. 100), others higher (Refs 142, 143), expression. Interestingly, in human infants, reduced IL-12 abundance might be a risk factor for NEC: Preterm infants with a low bioactivity IL-12p40 promoter polymorphism exhibited a higher risk of NEC (CTCTAA allele, OR 2.9, 95% CI 1.4–6.0, *P* = 0.004) compared with infants with homozygous IL-12 CTCTGC alleles (Ref. 144).

### IL-18

IL-18 is a pleiotropic cytokine with functions in innate and adaptive immunity. In concert with IL-12, IL-18 enhances IFNγ production and promotes Th1 differentiation (Ref. 145).

In experimental NEC, IL-18 appears to aggravate the disease process. Ileal IL-18 protein abundance increased progressively with severity of NEC injury in rats (Fig. 2e) (Refs 143, 146). Furthermore, IL-18-deficient mice were partially protected from NEC injury (Ref. 147), and the protection of anti-TNF treatment was associated with reduced intestinal IL-18 protein (Ref. 101).

However, the available human evidence disagrees with the animal findings. Ileal *IL18* mRNA was decreased in NEC infants compared with controls (Ref. 86). Similarly, a low-expression polymorphism (IL-18 A-607) was more frequent in infants with stage III NEC than in those with stage I/II (Ref. 148), and plasma IL-18 was moderately reduced in ELBW infants who subsequently developed NEC compared with infants that did not (Ref. 149).

## Th2 Cytokines

Th2 cytokines studied in NEC include IL-4, IL-5, and IL-13. IL-4 is the signature cytokine of the Th2 subset as it promotes Th2 polarisation, suppresses Th1 responses, and induces B cell immunoglobulin class switching to IgE. The functions of IL-13 are similar to those of IL-4, including IgE class switching and activation of mast cells and eosinophils. IL-5 acts on eosinophils, promoting their activation, survival, and adhesion (Ref. 145). The intrauterine environment favours Th2 polarisation (Ref. 53).

Increased ileal IL-4 and IL-5 accompanies NEC progression in rats (Ref. 70). Similarly, in a small human study, PBMCs isolated from stage II and III NEC infants at diagnosis exhibited 3-fold more cells spontaneously secreting IL-4 than GA-matched healthy controls (Ref. 138). However, comparing pre-operative NEC infants and GA-matched controls, serum IL-4 was not different, while IL-5 was 50% lower (Ref. 88), a surprising finding as onset of NEC coincides with eosinophilia (Ref. 150). Moreover, infants affected by NEC less frequently carried a high-bioactivity variant of the IL-4Rα chain (Ref. 151).

A marked increase in ileal IL-13 in NEC rats occurred after onset of tissue injury (Ref. 70). Others have proposed that IL-13 protects the gut by curbing excessive IL-17 and limiting its colitogenic effects (Ref. 152). However, IL-13 also causes epithelial dysfunction such as goblet cell hyperplasia and mucus hypersecretion.

## Th17 Cytokines

The Th17 signature cytokine, IL-17A, has several pro-inflammatory effects that are important for host protection against extracellular bacteria, including induction of chemokines (CXCL1, CXCL6 and CXCL10) and neutrophil recruitment and activation (Ref. 145). IL-23 induces Th17 polarisation, stimulates IL-17A in effector T cells, and is necessary for differentiation and effector functions of Th17 cells. Dysregulation of the Th17 pathway has been linked to inflammatory bowel diseases such as CD and ulcerative colitis (Ref. 153).

Th17 responses likely also play a pathogenic role in NEC. For example, RNA sequencing has revealed remarkable similarities in the signalling pathways affected by NEC, CD and paediatric CD (Ref. 135). Lamina propria CD4^+^ Th17 cells were more than 2-fold more abundant in NEC mice compared with controls (Ref. 69), and intestinal IL-17A and IL-17 receptor A (IL-17RA) was increased in mouse and human NEC (Ref. 69). These observations are in agreement with formula-fed preterm NEC baboons who exhibit a 5-fold increase in ileal *IL17A* gene expression compared with GA-matched non-NEC preterm controls (Ref. 50), and with ileal *Il23* mRNA being 6-fold higher in NEC rats than in dam-fed controls (Refs 142, 154). Moreover, intraperitoneal injection of recombinant IL-17A in newborn mice led to loss of intercellular tight junctions in the villi, reduced enterocyte proliferation and increased crypt apoptosis (Ref. 69). The detrimental effects of IL-17A in murine NEC were mediated by IL-17R, as these effects were abrogated by blockade of IL-17R with an antibody (Ref. 69). Similarly, inhibition of STAT3, a critical mediator of T cell differentiation towards a Th17 phenotype, using the compound WP1066 was also protective against murine NEC; WP1066 reduced Th17 cells and increased Tregs (Ref. 69). In fact, the balance between Tregs and Th17 cells may be critical in facilitating NEC, as one of the consequences of TLR4 deficiency was restoration of the Treg/Th17 ratio and near complete prevention of the NEC-associated intestinal infiltration of CD4^+^ T cells (Ref. 69).

By contrast, systemic IL-17 was reduced in 21-day-old babies that subsequently developed NEC compared with infants that did not (Ref. 149). Likewise, there were 50% fewer of the Th17-associated intestinal intraepithelial γδ-T cells in the ileum of acute surgical NEC infants than in non-NEC controls (Ref. 155). Furthermore, expression of the Th17 transcription factor RAR-related orphan receptor C (*RORC*) was 10-fold less in the ileal mucosa of NEC infants compared to non-NEC controls (Ref. 155).

In summary, although a disease-promoting role for Th17 polarisation may be emerging (Ref. 69), the data from humans frequently contradict those from animals in the field of adaptive immunity in NEC, and there are only few mechanistic studies. Moreover, the possibility that different Th subsets may dominate during different NEC stages remains poorly studied; thus, current evidence does not allow a conclusion on the relevance of Th polarisation in NEC.

## Immunoglobulins

Immunoglobulins (Ig) are produced by B cells in five isotypes (IgA, IgD, IgE, IgG and IgM) and function as antibodies or receptors that target foreign invaders such as bacteria, viruses, fungi, parasites and toxins, assisting in their neutralisation in cooperation with other immune cells. Ig-mediated host defence in the gut is immature even in infants born at term and is thus temporarily dependent on Ig transfer from the mother (Ref. 156). Breast milk is a major source of Ig for the newborn infant and has been proposed as one of the major factors by which breast milk protects against NEC. The Ig content in infant formula is low or absent (Ref. 28).

Ig supplementation was suggested as a prophylactic for NEC, with two small human studies reporting successful reduction of NEC incidence in infants orally administered either IgG alone (Ref. 157) or a mixture of IgA and IgG (Ref. 158). However, in a larger study, oral supplementation of IgG alone did not reduce NEC incidence (Ref. 159). A systematic review of these studies concluded that IgG or IgG+IgA demonstrated no significant reduction in incidence of definite NEC, suspected NEC, need for surgery or death from NEC in preterm and LBW infants (Ref. 160). Similarly, a systematic review of intravenous immunoglobulin administration to preterm or LBW infants or both also did not find any statistically significant difference in the incidence of NEC (Ref. 161). Thus, current evidence does not support the administration of oral or intravenous Ig for the prevention of NEC.

## Chemokines

### IL-8

IL-8 is a member of the CXC chemokine family that is produced by a variety of immune and non-immune cells (Ref. 145). Its main effector role is to recruit neutrophils to the site of inflammation.

The premature human gut readily produces IL-8 (Ref. 162) and unlike in the adult immune system, IL-8 production is also a major T-cell effector function in preterm infants (Ref. 163). *IL8* mRNA expression was increased in intestinal resection specimens from NEC infants compared with non-NEC controls (Fig. 2e) (Refs 86, 164) and serum IL-8 holds substantial potential as a diagnostic marker for NEC (see the ‘Biomarkers’ section).

## Other mediators

### Transforming growth factor beta (TGF-β)

The biological activities of TGF-β are pleiotropic and strongly dependent on the target cell/organ and the local cytokine milieu. In the context of adaptive immunity, TGF-β may support anti- and pro-inflammatory responses, for example suppressing Th1- and Th2-polarisation and promoting Treg functions, but also inducing Th17 cell differentiation (Ref. 165).

However, there is good evidence that TGF-β-deficiency promotes NEC. Disruption of TGF-β-signalling via depletion of TGF-βRII significantly increased the severity of platelet-activating factor (PAF) + LPS-induced NEC injury in 10–12-day-old mice compared with controls (Ref. 166). Moreover, tissue damage was ameliorated by enteral TGF-β_2_-supplementation in the PAF + LPS model and in formula-, hypoxia-, and cold stress-triggered mouse NEC (Ref. 166). Likewise, oral administration of TGF-β_1_ to NEC rats resulted in moderate suppression of NF-κB activation in ileal IECs and was associated with a 20% overall reduction in NEC incidence compared with vehicle-fed controls (Ref. 167).

Intestinal TGF-β_2_ bioactivity, protein abundance, and gene expression were markedly reduced in NEC patients compared to GA-matched non-NEC controls (Fig. 2f) (Ref. 166) and preterm versus term infants (Refs 50, 166). A similar TGF-β_2_ deficiency was observed in the intestine of formula-fed preterm baboons, and was even more pronounced in preterm baboons with NEC (Ref. 50). In fact, the protective properties of human breast milk may in part be mediated by TGF-β_2_, which it contains in high quantities (Ref. 28).

Mechanistic studies showed that human adult PBMC-derived macrophages develop increasing LPS tolerance when exposed to media conditioned by increasingly mature intestines, an effect mediated primarily through TGF-β_2_ and to a lesser extent by TGF-β_1_ (Ref. 166). In the developing intestine, macrophage production of pro-inflammatory cytokines may thus be suppressed by TGF-β, promoting tolerogenicity to commensal bacteria (Ref. 166). This function combines with TGF-β_2_-mediated cytoprotection (Refs 168, 169), rendering TGF-β_2_ a key protective player in NEC.

### Platelet-activating factor

PAF is a pro-inflammatory phospholipid mediator that activates pathways such as protein-kinase C (PKC), mitogen-activated protein kinases (MAPK), phosphatidylinositol 3-kinase (PI3K) and NF-κB (Refs 170, 171).

Intravenous administration of PAF in adult rats causes NEC-like ischaemic necrosis in the small intestine (Ref. 172). Pre-treatment with a low dose of LPS further aggravates these lesions, suggesting a synergistic effect between TLR signalling and PAF in intestinal disease (Ref. 172).

Whereas a moderate elevation of circulating and stool PAF is physiological upon commencement of enteral feeding in newborn babies (Refs 173, 174), this increase is more pronounced in formula-fed infants. Even higher PAF concentrations were observed in NEC patients compared with non-NEC controls (Fig. 2e) (Refs 91, 174).

The human neonate has a reduced capability to control substantial increases in PAF as the activity of its degrading enzyme, PAF-acetylhydrolase (PAF-AH) remains low in the first few weeks of life (Fig. 2f) (Ref. 175). Unlike formula, breast milk contains PAF-AH, which likely contributes to breast milk-mediated protection from NEC (Ref. 176). Although postnatally the circulating PAF-AH concentrations are similar in term and preterm infants (Ref. 175), in the setting of NEC, PAF-AH activity is reduced by more than 50% compared with non-NEC controls (Ref. 91). PAF-AH-deficient mice were more than twice as likely to develop experimental NEC than wild-type mice and exhibited a significantly higher abundance of inflammatory mediators such as CXCL1 and inducible nitric oxide synthase (Ref. 177). A beneficial role of PAF-AH is supported by the demonstration that intravenously administered recombinant PAF-AH protected against experimental NEC injury (Ref. 178). Furthermore, blockade of the PAF receptor ameliorated NEC-associated tissue damage in rats (Ref. 179) and piglets (Ref. 180).

## Biomarkers

Among the major challenges clinicians face in caring for infants who may have NEC, unequivocal identification of the disease in its early stages, differentiating it from sepsis or spontaneous intestinal perforation (SIP), and deciding if and when to proceed with surgery, stand out. Identifying and validating biomarkers to guide clinical decision making would represent a major advance in neonatal medicine. A recent review summarised potential biomarkers in NEC (Ref. 181); here, we focus on promising candidates with a relevance to immunology.

The acute phase reactant CRP is widely used as a marker of inflammation in NEC and many other diseases. Whereas CRP is non-specific and cannot be used to differentiate NEC from sepsis, it may be useful to determine disease progression; for example, a persistently elevated CRP may be indicative of treatment failure, whereas normalisation may indicate success.

IL-6 may have greater sensitivity and specificity than CRP for charting NEC disease. In surgical NEC infants, serum IL-6 was up to 60-fold higher than in controls (Refs 88, 109, 110), and was correlated with disease severity. In small studies on blood samples obtained within 48 h of NEC diagnosis (Refs 94, 95), IL-6 was undetectable in stage I, whereas the mean concentrations were 127 pg/ml in stage II and 3127 pg/ml in stage III patients. Post-operatively, stage III patients exhibited a decline in serum IL-6 to stage II levels, and importantly, mean IL-6 was 3-fold higher in infants that subsequently died than in survivors (Ref. 95). Furthermore, pre-operative IL-6 concentrations were markedly lower in SIP, a condition that is sometimes difficult to differentiate from NEC (Refs 62, 182). Similarly, a mathematical model employing the sequential use of IL-10, IL-6 and RANTES plasma measurements predicted the development of disseminated intravascular coagulation in VLBW infants with severe sepsis and NEC (Ref. 183). Thus, IL-6 may assist clinicians in assessing NEC disease severity and progression, and in distinguishing between NEC and SIP, but not sepsis.

Several other biomarkers of immune function and intestinal injury have been suggested to predict the progress of NEC. Higher plasma and urinary abundance of I-FABP is correlated with more severe intestinal damage, and predicted the need for surgery (Refs 184, 185, 186). Similarly, increased serum IL-1Ra (>130 000 pg/ml) at NEC onset was 92% specific in identifying infants whose disease subsequently progressed to stage III (Ref. 89). Moreover, serum IL-1Ra (>60 000 pg/ml) at NEC onset was 100% specific and 68% sensitive in classifying patients as suspected (stage I) or definite (stages II and III) NEC (Ref. 89). NEC patients exhibited higher serum IL-8 than healthy infants and babies with sepsis and non-inflammatory intestinal conditions (Refs 88, 109, 149, 187).

Increased serum IL-8 at diagnosis of NEC predicted the need for surgery and correlated with 60-day mortality (Ref. 188). Pre-operative serum IL-8 moreover predicted subsequent NEC severity, with 20-fold more IL-8 in pan-intestinal than in focal NEC cases (2750 versus 171 pg/ml) (Ref. 189). Furthermore, compared with pre-operative abundance, serum IL-8 dropped by 60% in focal NEC, by 92% in multifocal NEC, and by 96% in pan-intestinal NEC by post-operative day 3 in infants that survived the disease; note there were no postoperative data on non-survivors, as most of them died within 24 h (Ref. 189). Like IL-6, IL-8 also differentiated NEC from SIP (Ref. 182).

Deficiencies in anti-inflammatory mediators such as TGF-β_1_ and inter-alpha inhibitor protein (IaIp) may serve as predictive biomarkers for NEC onset. ELBW infants who subsequently developed NEC exhibited low circulating TGF-β_1_ from the first day of life, a concentration <1380 pg/ml predicted 64% of NEC cases (Ref. 149). Similarly, low plasma IaIp differentiated between NEC and non-specific abdominal disorders (Ref. 190).

Stool measurement of calprotectin also has potential for predicting NEC onset and severity (Refs 191, 192, 193). However, larger studies are required to resolve the wide variations in calprotectin concentrations in faecal matter and to establish universal thresholds for NEC diagnosis (Ref. 181).

It is common practice to combine several biomarkers into scores to achieve maximal diagnostic and predictive power. An example in NEC is the combined use of serum amyloid A and apoplipoprotein CII (ApoSAA score) that can guide the decision to initiate antibiotic treatment, as the ApoSAA score stratifies infants into low- and high-risk groups for sepsis/NEC (Ref. 194). The LIT [liver-fatty acid binding protein, intestinal-fatty acid binding protein (I-FABP) and trefoil factor-3] score can be used to determine NEC from sepsis and, more importantly, to differentiate the need for surgery and predict chance of survival (Ref. 195).

## Clinical trials

No immune mediator or inhibitor has been tested for efficacy in treatment of NEC. The clinical trials landscape is dominated by probiotics, which are thought to restore the gut flora to its healthy, diverse state, thus indirectly modulating the preterm gut immune system towards its tolerogenic poise.

## Conclusion and Outlook

Although the current data paints a complex picture of the vicious disease cycle in NEC (Fig. 2), two features stand out. First, there is a clear link between marked increases in certain pro-inflammatory mediators, including TLR4, TNF, IL-18, IFNγ, PAF, IL-6, IL-8, IL-1β, NF-κB and possibly IL-17A, in intestinal tissue on the one hand, and increased NEC severity on the other. Thus, beyond confirming that NEC occurs in the setting of excessive inflammation, research should focus on the aforementioned mediators in order to identify potential therapeutic targets. Second, it is likely that deficiencies in protective mediators such as TLR9, IL-1R8, IL-1Ra, TGFβ_2_, PAF-AH, and IL-10, as well as in Tregs, permit development of excessive inflammation in NEC, and thereby predispose infants to the disease.

On the biomarker front, immune mediators such as IL-1Ra, IL-6, IL-8, and TGF-β_1_ have emerged as promising candidates. Measurement of gut-specific markers such as I-FABP also demonstrates potential for management of NEC. Before clinical implementation, these observations need to be confirmed in larger multicentre trials.

At present we have insufficient evidence to draw a conclusion on the involvement of adaptive immunity in NEC initiation and perpetuation; however, recent data on a pathogenic role of Th17 responses and a Th17/Treg imbalance invite further exploration. On a cautionary note, findings in animal models and in the human have frequently proven contradictory, pointing to the danger inherent to relying too heavily on animal work. Furthermore, increasing the use of ‘omics’-approaches in NEC research will identify yet unknown mediators that contribute to NEC pathogenesis, and studies addressing the contribution of different cell types to the disease (e.g. IEC versus macrophages versus lymphocytes) are needed. The large datasets that are becoming increasingly available should also be mined in search for abnormalities such as SNPs or other genetic variants relevant to NEC.

While we have in recent years made progress in understanding some aspects of NEC, it is clear that a major research effort is required if an immunology-based treatment for NEC is to emerge. Only such an effort can banish the spectre of NEC, which looms over NICUs and continues to kill preterm infants.

## Further reading, resources and contacts

http://www.morgansfund.org/

The Morgan Leary Vaughan Fund for Necrotizing Enterocolitis (NEC) is an all-volunteer, public charity dedicated to promoting public awareness about NEC and the potentially devastating effects it can have on preemies and their families, and to advancing research to prevent, diagnose, treat, and ultimately, cure NEC.

http://necsociety.org/

The NEC society is a non-profit organisation that seeks to raise awareness of the risk factors of NEC in the wider community, advocate for better policies and practices to best protect preterm infants from NEC and assist in encouraging future NEC research.

http://www.preemieparentalliance.wildapricot.org/

The Preemie Parent Alliance represents a diverse set of organisations that provide information, support and resources to parents of preterm infants.
